# Southern Medical College

**Published:** 1889-09

**Authors:** 


SOUTHERN MEDICAL COLLEGE.—See Southern Medical College advertsement on page 12, advertising department, and special notice on page 359, reading department.

